# Prevalence and antimicrobial resistance profiles of Neisseria gonorrhea and Chlamydia trachomatis isolated from individuals attending STD clinics in Kampala, Uganda

**DOI:** 10.4314/ahs.v22i3.8

**Published:** 2022-09

**Authors:** Edith Nakku-Joloba, Gerald Mboowa, Willy Ssengooba, Anthony Kiyimba, Edgar Kigozi, Hannington Baluku, Lucy Alinaitwe, Ronnie Nyote, Jupiter Marina Kabahita, Paul Mutumba, Fred A Katabazi, Noah Kiwanuka, Nelson Sewankambo, David P Kateete

**Affiliations:** 1 Department of Epidemiology and Biostatistics, School of Public Health, Makerere University College of Health Sciences. P.O. Box 7072, Kampala, Uganda; 2 STD Clinic/Ward 12 Mulago National Referral Hospital; 3 Department of Medical Microbiology, School of Biomedical Sciences, Makerere University College of Health Sciences. P.O. Box 7072, Kampala, Uganda; 4 Department of Immunology and Molecular Biology, School of Biomedical Sciences, Makerere University College of Health Sciences. P.O. Box 7072, Kampala, Uganda; 5 Department of Medicine, and Clinical Epidemiology Unit, Makerere University College of Health Sciences. P.O.Box 7072, Kampala, Uganda

**Keywords:** Chlamydia trachomatis, Neisseria gonorrhoeae, Prevalence, Antimicrobial resistance, Genomic Analysis, Sexual partner networks

## Abstract

**Background:**

Sexually transmitted diseases (STDs) management in sub-Saharan Africa is syndromic but molecular diagnostics provide quicker, sensitive diagnosis and treatment. Effective STD control hinges on identification and treatment of infected persons and sexual partner contact tracing.

**Objectives:**

This study assessed feasibility of using the Xpert CT/NG test to identify prevalent Chlamydia trachomatis (CT) and Neisseria gonorrhea (NG) infections among STD clinic attendees and their sexual partners and tested for antimicrobial resistance for N. gonorrhea.

**Methods:**

A cross-sectional study was conducted at 4 outpatient STD clinics in Kampala, Uganda from February 2019 to October 2019. Participants received a syndromic diagnosis, were tested for NG and CT, as well as their sexual partners. Urine (men) and high vaginal swabs (women) were collected, examined using Xpert CT/NG assay. A total of 79 participants were enrolled at baseline of whom 25 had CT/NG. 21 partners of infected baseline participants and 7 partners of the 21 primary partners were enrolled.

**Results:**

The mean age of the reported sexual partners was 26 (18–43) years. The prevalence of NG was 25% at baseline and 18 % for CT. Nine (11.4%) people were dually infected. Men were more likely to have NG (p<0.001) at multivariable level. Two participants tested HIV-1 positive. On microbiological culture, 8 samples (2.5%) grew NG and all were resistant to penicillin, ciprofloxacin. For CT, we found a preponderance of the F-serovar in this population.

**Conclusion:**

The most prevalent organism was Neisseria gonorrhea. Generally, the prevalence of CT and NG was high. Infection proportions increased among primary partners, particularly women. Etiologic testing without partner tracing and treatment may underestimate burden of CT/NG in this population and contribute to re-infection.

## Introduction

Chlamydia trachomatis (CT) and Neisseria gonorrhea (NG) are sexually transmitted infections (STIs) that continue to be an indispensable risk factor in the global spread of HIV. Globally, more than 1 million curable STIs occur each day. According to the World Health Organization (WHO) global estimates for 2016, there were roughly 376 million new infections of the four curable STIs – Chlamydia, Gonorrhoea, Syphilis and Trichomoniasis^1^. In Uganda, the prevalence of NG and CT is 5.4% and 0.9% respectively, and having primary or less education for both participant and spouse was associated with increased risk of acquiring STIs^2^. Studies carried out in sub-Saharan Africa, a region hit hardest by HIV, indicate that NG and CT increases HIV acquisition and transmission^3^–^8^.

The inflammatory process resulting from STIs increases viral shedding of HIV-1 in genital tract^9^–^11^, increasing the risk of HIV-1 transmission sexually^2^. Through whole-genome sequencing, NG has been found to have local, national, and international transmission^12^. Therefore, accurate early diagnosis and subsequent treatment of these STIs is imperative in the global fight against STIs. We conducted a baseline study among persons with complaints of genital discharge attending several out-patient STI services clinics in Kampala, Uganda, aiming to determine the prevalence of NG and CT using the Cepheid GeneXpert CT/NG and the antimicrobial susceptibility profiles of isolated NG. The Cepheid GeneXpert CT/NG is a new Food and Drug Administration (FDA)-approved rapid molecular assay for simultaneous detection of N. gonorrhoeae and C. trachomatis. This assay is the first genetic point-of-care assay that amplifies one chromosomal target (CT1) for the detection of C. trachomatis, two chromosomal targets (NG2 and NG4) for detection of N. gonorrhoeae, a single-copy human gene which should be present in each specimen to act as a sample adequacy control (SAC), and Bacillus globigii DNA added to each cartridge to serve as a sample-processing/internal control (SPC)^13^.

## Methods

Using a population prevalence from previous studies and the conservative approach (prevalence of 0.05 for NG and 0.025 for CT), for a desired 95% confidence interval, and 5% error and calculating for non-response rates, the calculated sample size for the baseline was 79. Consenting persons aged 18 years and above with complaints of genital discharge attending 4 clinics providing STI services in Kampala, Uganda were enrolled between February 2019 and August 2019. Interviewer-administered questionnaires were used to collect data on socio-demographic characteristics, descriptions of partner sexual networks and other factors hypothesized to be associated with NG and CT infection. A syndromic diagnosis, based on national guidelines, as made by a clinician. Blood and swab samples were taken from female participants as well as high vaginal swab (HVS) while urethral swabs and urine samples were taken from male participants. Treatment was provided at first contact as per national guidelines.

### Participants' enrollment, sexual partners and follow-up

Consenting participants provided their locator information as well as contacts of their sexual partners. Enrolled participants chose to contact partners directly or with clinician involvement. Participants had the following tests done; Xpert NG/CT test, HIV and Syphilis, culture and antibiotic sensitivity testing on modified Thayer-Martin agar (Oxoid, UK) for NG. All persons who had a positive NG Xpert test had a culture done on their sample and E-test for drug sensitivity of the bacteria. Individuals with reported discharge were treated according to the Uganda STD National Treatment Guidelines Algorithms^14^. Those who later tested positive for resistant CT/NG infection(s) received appropriate treatment. All participants who tested positive were followed up after two weeks to assess cure and were found to be culture-negative. Further, data was collected on other sexual behavior, alcohol use and substance abuse using the Substance and Drug Abuse World Health Organization tool^15^. Reported known partners that participants were willing to provide information about, were contacted and offered testing and treatment. Partners of infected participants were identified by their enrolled partners and invited, then followed up by study staff in collaboration with the enrolled partners.

### Sample collection and transportation

Vaginal swabs were collected from women using Dacron swabs while urine specimen collection kits were used to collect urine from symptomatic males. and urethral swabs to collect urethral swabs from men. One swab was used for immediate testing of CT/NG using the Xpert assay while the second swab (placed in Amies transport media in cold box) was used for culturing of NG. Swab samples from men and women were sent to the clinical microbiology laboratory for NG culturing and sensitivity testing at the department of medical microbiology, Makerere University college of ealth sciences. We tested the samples using Xpert® CT/NG assay at the Mycobacteriology (BSL-3) laboratory at the same department but without culture for CT since it is a fastidious organism. For NG tests, we did both Xpert testing and culture.

### Identification of NG and CT using the Xpert® CT/NG assay

Xpert® CT/NG assay was done for identification of CT/NG according to manufacturer's instructions (Cepheid, Inc.). Briefly, this involved two quick steps: 1) Urine or swab samples previously stored in the Cepheid transport reagent were retrieved; 2) 1.0 ml of the sample was transferred to the Xpert® CT/NG cartridge and loaded into the Xpert machine^16^.

### Isolation, identification and antimicrobial susceptibility testing of NG isolates

NG isolates were cultured from consecutive symptomatic gonorrhoea patients using standard microbiological methods. Briefly, the collected swabs were inoculated onto modified Thayer-Martin agar and placed in a candle jar with 5–10% CO2. The inoculated plates were incubated at 370 C for up to 72 hours. NG was identified based on characteristic colony morphology, Gram staining and biochemical properties. In this study, pure NG growth in any numbers was considered significant and were subsequently tested using the E-test for sensitivity. All gonococcal isolates were stored as part of routine diagnostics. The minimum inhibitory concentration (MIC; mg/L) for cefixime, ceftriaxone, penicillin G, azithromycin, ciprofloxacin, spectinomycin used were determined and interpreted according to the instructions from the manufacturer (bioMerieux SA, Marcy-lÉtoile, France), in accordance with the Clinical and Laboratory Standards Institute (CLSI (M100-S22).

### Ethical statement

Ethical approval was received from Makerere University School of Biomedical Sciences Ethics Committee (SBSREC 557), the Uganda National Council for Science and Technology (SS 2425). Administrative approval was received from Mulago Hospital IRB and the Kampala Capital City Authority directorate of Public Health and Wakiso district health office.

## Results

**Figure 1:**
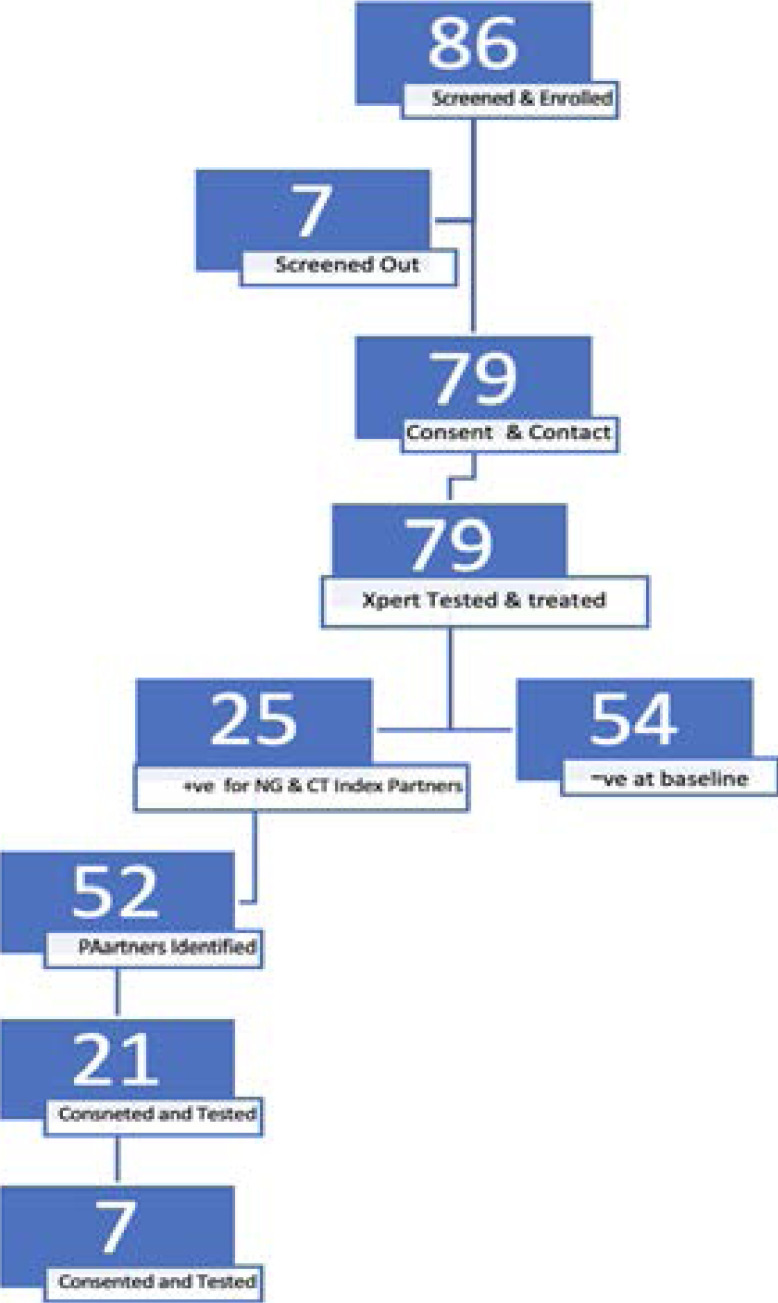
Study enrollment numbers

A total of 86 participants were screened at baseline, of these, 7 persons were not willing to give contacts to their partners and therefore were not enrolled. We enrolled 79 persons at baseline both male and females from 5 health centers. The sites where participants were enrolled; Most at Risk Populations Initiative - MARPI-STD clinic, Kasangati Health Centre IV, Komamboga HCIV and Kisugu HCIV-11. A total of 79 persons at baseline (baseline partners) were enrolled. In case the baseline person had a positive test for C. trachomatis or N. gonorrheae, then partners were contacted for enrollment. On follow up of the 25 identified positives, a total 52 partners were reported as sexual partners and invited to join the study and followed up by study clinicians. Out of these, 21 partners of baseline partners consented and enrolled into the study. We later enrolled 7 partners of these 21 enrolled primary partners (i.e. secondary partners) in the reported network. Of the 107 people enrolled; 79 were at baseline, 21 partners of baseline participants and 7 partners of primary partners. (Figure 1.) The study exhausted the resources to enroll willing sexual contacts at that point.

Total of 107 people; 35 (32.7 %) men and 72 women sexual partners were enrolled in this study as baseline, primary and secondary partners.

### Socio-Demographic Characteristics of Baseline participants

At baseline, of the 79 persons who made up the index participants, 59 (75%) were female and 20 (25 %) were male. The enrolled partners (primary partners) consisted of 21 persons, of whom 12 (57%) were female and 9 (43%) male. The median age of enrolled participants at baseline was 27 years (Inter quartile range IQR; 18 to 43). Participants reported an average of 1 child each (range of 0 to 5) and an average income of 250,000 Uganda shillings per month (estimated 66 US dollars)^17^. Participants had on average 9 years of education and had stayed a mean 5 years together with their main partner. Twelve (12) women of the 59 (20%) at baseline reported a known other partner apart from the main partner. Four (4) men of the 20 (20%) reported known other partners. A total of 9 (45%) men at baseline were circumcised while 42 (53%) females at baseline reported that their main partner was circumcised. Of the males, 7/20 (35%) were single while 23/59 (40%) of females were single. Participants reported an average of 2.4 sexual partners over the last 3 months.

### Sexual behavior

Most participants reported sexual activity. Majority (77, 95%) reported sexual activity in the last 3 months. Average age of sexual debut was 17.5 years (s.d=3.0), range (10–28). Participants reported an average of more than one sexual partner in the last 3 months. Most women, 53 (87%) reported one sexual partner. Two females reported female sex partners and 2 males reported male sexual partners at baseline. Two women reported having no sexual partner in the past 3 months. Eight (40%) men reported an average of 2 or more partners. while 5 (7%) women reported having 2 or more partners each. There were some outliers as 5 (7%) women reported an average of 3 men partners each compared to all other women thus skewing the data for women.

### Perception of STD and HIV risk

Participants reported that they perceived themselves at risk of acquiring STIs according to a number linear measurement of ‘own perceived risk’ scoring from 0 to 10 and based on a Likert scale. On the linear scale, 30 women out of 77 reported they felt at no risk for acquiring STIs at all (score 0) while 27 reported they perceived themselves at maximum risk for acquiring STIs (score 10).

### STD History

Forty (50%) of the females and 7 (35%) males reported history of STD in the last three months while 34 (43%) females and 7 (35%) males reported having sought for treatment for some STD in the last 3 months and received it. A total of 23 (39%) women and 6 (32%) men reported ever having been diagnosed with an STD by medical personnel in the past one month. A total 41 (43%) people reported they had been treated for genital discharge; of these 7 were men (8%) and 34 were women (82%). Twelve (12) people had ever tested for NG (7 women and 5 men) in the past. Only 2 people reported they had ever tested for CT.

### Prevalence of CT and NG among participants

The estimated proportion of NG infection among the baseline samples (primary partner level) using the Xpert NG test was 20 (32%) of 79 enrolled at baseline. The overall proportion of CT infection in the baseline samples was 14 (18 %). Nine individuals tested positive for dual infection with NG and CT at baseline, a prevalence of 11.4% for dual infection.

### Secondary-level and Tertiary-level partners

Further tests among 21 enrolled partner participants of the positive testing baseline participants showed that among 21 partners,10 (50%) individuals had a positive test for CT while 8 individuals tested positive for NG (38%) and 5 (24%) individuals were dually infected with CT and NG. For individuals at the primary partner level, the proportion infected was higher compared to the baseline samples. At baseline, 2 were HIV-1 positive and 1 was Syphilis positive by rapid test.

### Antimicrobial susceptibility profiles of NG isolates

When the positive samples for NG were plated on MTA, 8 samples grew. All 8 isolates were tested for antibiotic sensitivity and found resistant to penicillin and ciprofloxacin. They were all susceptible to azithromycin and spectinomycin.

### Genomic analysis of NG

We analyzed the genomic data for the NG swabs that grew on culture. Isolates grew on culture. Only QC was done using FastQC and only 6 isolates passed. We aligned and called variants using snippy bioinformatics software (https://github.com/tseemann/snippy); which is a rapid haploid variant calling and core genome alignment used and a genbank reference of NG FA 1090 (https://www.ncbi.nlm.nih.gov/genome/?term=FA_1090).

### Phylogenetic analysis of Neisseria gonorrhea among partners

In summary, 7 analyzable data samples were compared with reference sequences. Three clades were generated with 1100NK, 1243NK and 1517NK, 1448NK being closely related respectively.

### Molecular typing of Chlamydia trachomatis isolates

There was a preponderance of the F serovar. Six (6) of the identified serovars were F, in 5 the OmpA gene was not detected and the conclusion was that probably these could have been another species of chlamydia and in one the J serovar was detected as well as another where the D serovar was detected.

### Risk factors associated with /CTNG infection

One main factor was associated with NG; either being a man or a woman and gender. Women were two and a half times less likely to be infected with NG than men (OR; 0.032–0.402). The association of gender and NG seen at univariate was maintained at multivariable level (Table 4).

**Table 4:** Univariate & multivariable associations; CT, NG infection at baseline, n=79

	Xpert CT+ (n=79) Univariate	OR (95% (CI) Multivariable	p-value	Xpert NG+ (n=79) OR (95%CI) Univariate	Multivariable
Age More Than 25=0	1.85(0.58–6.14)	1.4 (0.64–3.06) *	NS	1.59 *(0.75, 3.35)	NS
Gender Female=0	1.85(0.54–6.37)	0.52 (0.12 2.14) *	NS	0.11 (0.03–0.40) *	p<0.001
Education>7=0	1.16(0.35–3.90)	NS	NS NS	NS	NS
No. of Partners >2	1.56(0.16–16.51)	NS	NS	3.16 (0.41–24.11)	NS
Past STD History3m Yes=1	0.29(0.08–0.97)	NS	(p=0.07)	0.178 (0.059–0.540)	2.86 (1.53, 5.35)
Condom Use	(2%)	0.37 (0.10–1.3)+	6 (6%)	0.68(0.20–2.29)+	
Perceived HIV Risk High (5–10)	0.55(0.11–2.7)	NS	NS	0.88(0.25, 3.10	NS
Reports >1 partner	1.3(0.30–5.60)	NS		3.57 (0.90–14.1), 5.74)	NS
Perceived STD Risk Level	2.33(0.69–7.95)	NS		0.88 (0.16, 4.78)	NS

CT=*C. trachomatis* Infection, NG=*N. gonorrhea* infection, NS= Not statistically significant, +=positive.

## Discussion

This study found prevalence of CT of 18% (25% men, 15% women) and a prevalence of NG of 25% (55% men, 15% women) in STD clinic attendees in 4 clinics in and around Kampala, Uganda. The study found a proportion of 38% for CT (22% in men and 50% in women) among enrolled partners (higher). Prevalence of NG infection was similarly high among partners; 33% in partners (22% in men, 42% in women). Study found prevalence of dual infection at 13% at baseline increasing to 24% at primary partner level. This is among the few studies documenting etiological prevalence of CT and NG in Uganda using nucleic amplification tests (Xpert CT/NG). The prevalence of each CT/NG is higher than rates previously reported in Uganda. Katusiime et al reported a prevalence of NG at 0.3% among HIV-positive individuals attending an urban clinic in Uganda in 2011/2012^18^; their much lower prevalence could be explained in that their study diagnosed the infection microbiologically (as we did in the 8 samples that grew NG in this study). Further, study participants in that study were HIV-infected individuals probably receiving good care at clinics regularly.

Kakaire et al reported a prevalence of NG at 5.4% among Women living with HIV/AID; a high risk group for STDs^2^, their study utilized an in-house PCR to detect CT in specimens. This offers greater sensitivity than microbiological detection and may account for the higher prevalence observed in that study, but our study documented a value much higher than Katusiime et al. Our study has reported CT prevalence at 13 %, eight times higher than reported by Kakaire et al at 0.9% among PLWHA. This is probably because the Xpert® CT/NG assay is more sensitive than the in-house PCR used by their study and secondly the two different study populations may account for the observed variation in the prevalence.

A study by Mabonga and colleagues found a prevalence of 5.7% for NG in men and women in HIV clinics in Uganda^19^ higher than the Kakaire study but still less than in our study. Mabonga's study showed higher prevalence of gonococcal infection in men. The findings in our study are similar to this and earlier studies that have shown higher prevalence in men than women for gonococcal infection. Men tend to have higher prevalence because of their physiology which renders the presentation of NG in men to be more overt^20^. Targeted efforts are needed for men for themselves and for prevention of transmission to their partners. The high prevalence found in women in this study at baseline (15%) and higher at partner level (22%) means that treatment is critical too for women, especially because the course of gonorrheal disease is more chronic in women. The higher than usual prevalence of Chlamydial infection in women (15%) at baseline suggests there may be a lot of unrecognized STD in women in this population. The high numbers imply a possible public health epidemic that needs to be urgently managed.

This study is one of the earliest to document high etiologic prevalence of CT/NG infections in women in Africa in regular STD clinic populations. A study by Remco and colleagues found an average of 2.5% prevalence for NG and 5.5 % prevalence for CT in high risk women in rural South Africa^21^. The study illustrates the importance of development of more sensitive and faster diagnostic NAAT tests like the Xpert CT/NG that provide better diagnosis and more targeted treatment of CT/NG. There are many Xpert machines and other NAAT platforms in many laboratories across Uganda for TB testing^22^; the same platforms can take CT/NG cartridges for STD testing, making targeted treatment more feasible for CT/NG. This study's finding of high prevalence of CT and NG makes these resources even more important.

## Figures and Tables

**Table 1:** Baseline characteristics of participants in the CT/NG STD partner network

	Women (n=59)	Men	C95% CI), p-value
Sex	(75%)	(25%)	5 (36%)
			9 (64%)
Age (in years)			
Median, Range	26 (18–43)		
25 and below	23 (39 %)	05 (25%)	(1.5–14.00), .009*
Above 25	36 (44%)	15 (75%)	
Education, years <7 years	7 (4,10) 25 24	7 (2.2,9) 02 18	7 (6.2,8.8) 0.013 (chisq)
Any income			
>300000	31 (79%)	13 (73%)	(0.070–0.762), p=0.31
<300k	13 (21%)	05 (27%)	
Marital Status			
Married	32 (46%)	13 (65%)	0.79
Single	23 (54 %)	7 (35%)	
Average number Of children with partner	(0,5) 1.2 (sd1.4)	(0,5) 1.7 (1.4)	F=1.671 (p=0.200)
Children with partner (0,1) Mean (sd)	1.3(s.d=1.4)		
Number of current partners 0 1 2 3 4 Age at First Sex	2 52(87%) 04(7%) 00(0%) 1 17(sd= 2.8)	1 11(70%) 8(20%) 01(10%) 18.0 (sd=2.5)	<0.001 0.414
Number of sex acts with partner/week Median (Range) Mean (sd)	1.0 (0, 20) 2.9 (sd=4.4)	2.2 (s.d=3.7)	0.5 (0, 13) 0.095 (Chisq)
Age of Sexual Debut			
Median age (Range)	17.0 (10–28)	19 (20–24)	0.9

**Table 2:** Genomic analysis of NG isolates

	Sample_ID	Genomic Resistance	Sequence Type
A	1243NK	Penicillin-(*blaTEM*), *Tet*racycline-*Tet*(M), Cipro-(*gyrA*)	14451
B	1100NK	Penicillin-(*blaTEM*), *Tet*racycline-*Tet*(M), Cipro-(*gyrA*)	14451
C	1092NK	Penicillin-(*blaTEM*), *Tet*racycline-*Tet*(M), Cipro-(*gyrA*)	Unknown
D	1517NK	Penicillin-(*blaTEM*), *Tet*racycline-*Tet*(M), Cipro-(*gyrA*)	Unknown
E	1375NK	Penicillin-(*blaTEM*), *Tet*racycline-*Tet*(M), Cipro-(*gyrA*)	8111
F	1448NK	Penicillin-(*blaTEM*), *Tet*racycline-*Tet*(M), Cipro-(*gyrA*)	8162
G	1371NK	Penicillin-(*blaTEM*), *Tet*racycline-*Tet*(M), Cipro-(*gyrA*)	1932

**Table 3:** Microbiological analysis of CT isolates

Sample ID	Patient ID	Genotype/Serovar	Comment
1.CT001 (RPT?)	MA005-01-001-0	F (Shared with Partner)	Chlamydia trachomatis
2.CT003	MA005-01-001-0	F	Chlamydia trachomatis
3.CT004	MA007-0301-001-1	G	Chlamydia trachomatis
4.CT019	MA-004-0401-000-1	F (Shared with Partner 005)	Chlamydia trachomatis
5.CT024	KA-019-16-00-000-0	F	Chlamydia trachomatis
6.CT028	KA-026-23-00-0002-0	F	Chlamydia trachomatis
7.CT031	MA-005-01-01-001-0	F	Chlamydia trachomatis
8.CT043	KA-034-30-00-000-0	F	Chlamydia trachomatis
9.CT052	KA-043-37-00-000-1	OmpA gene not detected	Could be another species of Chlamydia
10.CT044	KA-049-40-00-000-0	OmpA gene not detected	Could be another species of Chlamydia
11.CT076	KO-064-48-01-001-0	OmpA gene not detected	Could be another species of Chlamydia
12.CT054	KA-046-20-01-001-0	F	Chlamydia trachomatis
13.CT57	KO-057-48-00-000-0	F (Partner of 064)	Chlamydia trachomatis
14.CT70	KO-067-51-01-001-0	J	Chlamydia trachomatis
15.CT73	KO-071-51-02-001-A-0	D	Chlamydia trachomatis
16.CT081	KA-047-37-01-001-0	F	Chlamydia trachomatis
17.CT078	KA-070-60-02-001-A-0	F	Chlamydia trachomatis
18.CT068	K0-072-51-01-002-0	D	Chlamydia trachomatis
19.CT040	KA-042-36-00-000-0	OmpA gene not detected	Could be another species of Chlamydia
20.CT047	K0-051-42-00-000-0	OmpA gene not detected	Could be another species of Chlamydia

Each isolate harbored more than 100 variants showing that there is a lot of diversity in these strains.
